# Kounis Syndrome Type 2 due to Ingestion of a Fish Meal: A Case Report

**DOI:** 10.1002/ccr3.70209

**Published:** 2025-03-27

**Authors:** Bassem Al Hariri, Obada Adel Alsakaji, Abdelkarim Mohamed, Muad Abdi Hassan

**Affiliations:** ^1^ Department of Medicine Hamad Medical Corporation Doha Qatar; ^2^ College of Medicine Qatar University Doha Qatar; ^3^ Medical Education Department Hamad Medical Corporation Doha Qatar

**Keywords:** allergic angina syndrome, histamine, hypersensitivity, Kounis syndrome, myocardial infarction

## Abstract

Kounis syndrome is a type of acute coronary syndrome that occurs when a chemical mediator produced by mast cell activation leads to coronary artery vasospasm. This can happen due to anaphylaxis, anaphylactoid reactions, allergies, or hypersensitivities. There are three variants of Kounis syndrome, including vasospastic allergic angina, an atheromatous disease with plaque eruption, and stent thrombosis. In this case, the patient developed type 2 Kounis syndrome after consuming a fish meal, which caused him to present with a non‐ST elevation myocardial infarction (NSTEMI) at our hospital.

**Summary**

- Kounis syndrome, while not uncommon, is often underrecognized in medical literature. Healthcare professionals must identify this condition swiftly, as it necessitates immediate and decisive treatment.
- Prompt recognition and effective management—employing anti‐ischemic, anti‐thrombotic, and anti‐allergic therapies—can greatly enhance patient outcomes.
- Additionally, for select patients, coronary angiography should be considered an essential part of the management strategy.

**Abbreviations**
ACS: acute coronary syndrome
ECG: electrocardiogram
Echo: echocardiogram
EF: ejection fraction
IHSS: ischemic heart scombroid syndrome
LCX: Left Circumflex artery
NSTEMI: non‐ST‐segment elevation myocardial infarction
PCI: percutaneous coronary intervention

## 1 Introduction

Acute coronary syndrome (ACS) or allergic angina, which is caused by mast cell activation and accompanied by an anaphylactic response, is known as Kounis syndrome (KS) [1]. Anaphylaxis, anaphylactoid response, allergies, or hypersensitivity can all cause mast cell activation. Histamine, cytokines, and leukotrienes—some inflammatory mediators—are often activated in this process [2]. Various medications, foods, environmental exposure, and intracoronary stents might all be triggering factors [3]. Kounis syndrome has three types. Type I affects people with normal coronary arteries, which happens in normal coronary arteries without elevated cardiac enzymes, while Type II affects those with atheromatous disease with plaque eruption. Type III includes patients with coronary or stent thrombosis, with two subtypes: thrombus within the stent, subtype IIIa, and restenosis of the stent, subtype IIIb [4, 5, 6]. Here is a case with a likely instance of coronary vasospasm brought on by an allergic response after consuming food. However, his elevated level of troponin, with clinical presentation of an allergic reaction, chest tightness, and changes in the ECG, considered coronary angiography procedure him.

## 2 Case Presentation

**FIGURE 1:**
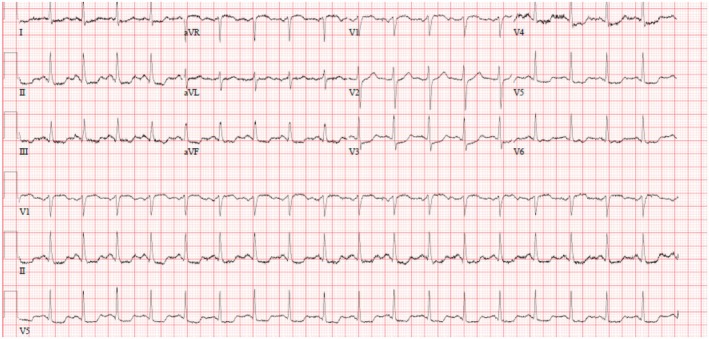
ECG Kounis syndrome type 1; Sinus Tachycardia; ST depression in II, III, aVF, I, V3–V6, and ST elevation in aVR.

A 41‐year‐old gentleman with a medical history of hypertension, diabetes mellitus, and appropriately treated rheumatic fever presented with complaints of generalized pruritus, redness, chest pain, palpitation, and giddiness for one day. His symptoms started 20 min after eating fish. Initially, the patient developed generalized pruritus when he noticed red spots all over his body. Then, it was followed by chest pain, palpitation, and giddiness. His pain was felt in the center of the chest as a heaviness or squeezing pain in nature, moderate to severe intensity, non‐radiating, with no aggravating or relieving factors. The patient denied a history of allergy to any food or medication. There was no significant finding on examination, and his vital signs were presented in Table 1. His initial labs showed elevated levels of troponin 460 ng/L, HbA1C of 6.6, and the rest of his labs were insignificant (Table 2). Initial Electrocardiogram (ECG) was significant for Sinus Tachycardia; ST depression in II, III, aVF, I, V3 –V6, and ST elevation in aVR (Figure 1).

**TABLE 1:** Vital signs readings.

Vital signs	At admission	After 3 days from the admission and after the PCI	At the discharge	Normal values
Temperature	36.6°C	36.7°C	36.5°C	35.5°C–38.5°C
Pulse rate	96 bpm	66 bpm	72 bpm	50–120 bpm
Respiratory rate	18 br/min	18 br/min	20 br/min	12–24 br/min
Systolic blood pressure	120 mmHg	114 mmHg	110 mmHg	100–180 mmHg
Diastolic blood pressure	70 mmHg	78 mmHg	66 mmHg	60–90 mmHg
Mean arterial pressure	87 mmHg	90 mmHg	81 mmHg	70–100 mmHg
SpO_2_	96%	98%	99%	

**TABLE 2:** Laboratory test results.

Lab tests	At the admission	After 3 days from the admission and after the PCI	At the discharge	Normal values
WBC	14.8 × 10^3^/μL	12.0 × 10^3^/μL	11.0 × 10^3^/μL	4–10 × 10^3^/μL
RBC	5.0 × 10^6^/μL	5.6 × 10^6^/μL	5.4 × 10^6^/μL	4.5–5.5 × 10^6^/μL
Hb	14.2 g/dL	15.9 g/dL	15.3	13–17 g/dL
Hct	41.1%	46.5%	45.2	40%–50%
MCV	82.0 fL	83.3 fL	83.1	83–101 fL
MCH	28.3 pg	28.5 pg	28.1	27–32 pg
MCHC	34.5 g/dL	34.2 g/dL	33.8	31.5–34.5 g/dL
RDW‐CV	12.3%	12.6%	12.6	11.6%–14%
Platelet	276 × 10^3^/μL	308 × 10^3^/μL	295	150–410 × 10^3^/μL
PDW	11.4 fL	10.7 fL	H 11.9	9.4–10.6 fL
Prothrombin time	11.6 s			9.4–12.5 s
INR	1.0			> 4.9 critically high
APTT	27.6 s			25.1–36.5 s
Urea	4.6 mmol/L	7.3 mmol/L	6.8 mmol/L	2.5–7.8 mmol/L
Creatinine	97 μmol/L	89 μmol/L	84 μmol/L	62–106 μmol/L
Sodium	142 mmol/L	138 mmol/L	138 mmol/L	133–146 mmol/L
Potassium	4.4 mmol/L	4.2 mmol/L	4.7 mmol/L	3.5–5.3 mmol/L
Chloride	105 mmol/L	105 mmol/L	103 mmol/L	95–108 mmol/L
Bicarbonate	23 mmol/L	22 mmol/L	22 mmol/L	22–29 mmol/L
Calcium	2.26 mmol/L			8.6–10.3 mg/dL
Magnesium	0.83 mmol/L		0.88 mmol/L	0.70–1 mmol/L
Bilirubin	9 μmol/L			0–21 μmol/L
Total protein	72 g/L			60–80 g/L
Albumin	37 g/L			35–50 g/L
Alkaline phosphatase	46 U/L			40–29 U/L
ALT	18 U/L			0–41 U/L
AST	25 U/L			0–40 U/L
CRP	< 2 mg/L			0–5 mg/L
Troponin	461 ng/L	220 ng/L	168 ng/L	3–15 ng/L
Cholesterol	5.0 mmol/L			5.2–6.2 mmol/L
Triglyceride	0.7 mmol/L			< 1.7–2.2 mmol/L
HDL	1.0 mmol/L			> 1 mmol/L
LDL	3.6 mmol/L			2.59–3.34 mmol/L
HbA1C	6.6%			< 5.7%–6.4%

### 2.1 Methods (Diagnosis, Investigations, and Treatment)

**FIGURE 2:**
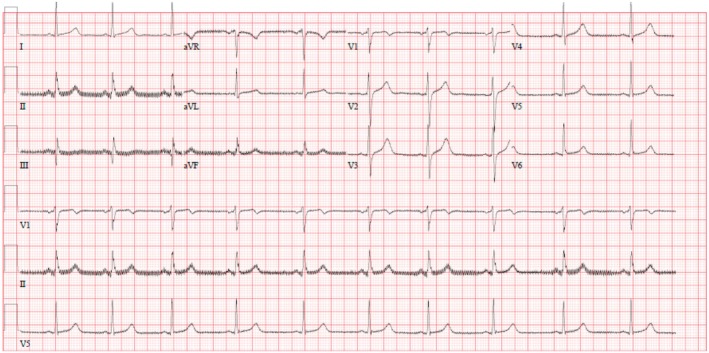
Sinus rhythm with short PR and ST elevation in inferior leads MI; II, III, aVF.

Further investigation was done with an echo, which showed normal right ventricle function, left ventricular EF of around 58%, and no regional wall abnormalities. After a while, from his admission, another ECG was taken. Compared with the first one, the second ECG showed some features of myocardial infarction with Sinus rhythm with short PR and ST elevation in Inferior leads (II, III, aVF) (Figure 2).

### 2.2 Conclusion and Results (Outcome and Follow‐Up)

**FIGURE 3:**
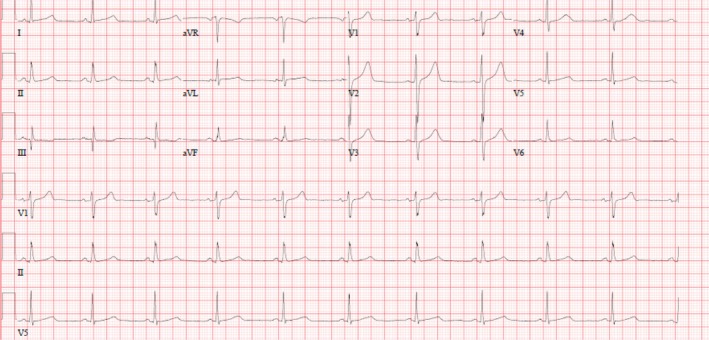
Normal ECG.

Due to raised troponin and the clinical history and presentation of the patient, we could not exclude Acute Coronary Syndrome. Therefore, the patient was admitted, started on full anti‐ischemic medication, and underwent a coronary angiography procedure that revealed Obstructive Single Vessel Disease in the Proximal LCX (Left Circumflex artery) and was treated accordingly. After that, his condition improved, his parameters settled down, and the ECG that was done afterward was normal (Figure 3).

## 3 Discussion

The term “Kounis syndrome” refers to the acute coronary syndromes brought on by mast cell activation, such as allergy or anaphylactic assaults. There are three types of this syndrome: type I variant, which affects people with normal coronary arteries and may be a sign of microvascular angina or coronary vasospasm in which the acute release of inflammatory mediators occurs with or without elevated levels of cardiac enzymes and troponin. Type II variant affects people with an atheromatous disease that is the culprit but is quiet, and where an allergic reaction can cause plaque erosion or rupture and acute coronary syndrome [4]. In addition to that, patients with coronary thrombosis or stent thrombosis who have stents in situ are included in the third variety or type III of Kounis syndrome [5]. There are two subtypes of type III: subtype IIIa, which is thrombus formed within the stent, and subtype IIIb, which is restenosis of the stent [6]. Mast cell degranulation and chemical mediator release such as histamine are linked to allergic and anaphylactic responses. The majority of the body's tissues, including the coronary arteries, include mast cells that can produce histamine and leukotrienes. Leukotrienes and histamine both have potent coronary vasoconstrictors. Furthermore, it is understood that smooth muscle cells' H1‐histamine receptors are enabled by histamine in coronary arteries to have a vasoconstrictive effect. Thus, histamine in the coronary arteries may cause coronary vasospasm during an allergic episode [7, 8, 9]. Acute coronary syndrome and decreased coronary blood flow may be caused by plaque rupture and thrombosis.

Several allergens have been reported to trigger Kounis syndromes, such as drugs, food, and contrast media [10]. That pathophysiology causes anaphylactic events with the participation of heart tissue, leading to triggered tachycardia, coronary vasoconstriction with impaired contractility, and atrioventricular (AV) conduction block [6, 11].

Our patient had risk factors for coronary artery disease, and we suspect that he suffered from allergic vasospastic angina. Because of his history of allergy and the allergic reaction he experienced, he is at a high risk of developing acute coronary syndrome after anaphylaxis. He mentioned eating fish, which may have triggered the allergic reaction by mediating with histamines and causing mast cell activation. Initially, he had ST depression on ECG, but later, it changed to ST‐elevation myocardial infarction due to his history of coronary artery disease. The treatment involved the use of antihistamines and a coronary angiogram for the single‐vessel obstruction.

Based on our patient's presentation, the possible diagnoses would be Kounis syndrome and Ischemic Heart Scombroid Syndrome (IHSS). The patient's medical history includes hypertension, diabetes mellitus, and past rheumatic fever, which was treated appropriately. The ECG changes suggest Kounis syndrome type 2, which is an allergic reaction caused by mast cell activation. However, it was unclear whether the fish meal the patient had was a new or a regular one or even canned fish food. To differentiate between these possibilities, we need to look at parameters such as allergic reactions, coronary artery disease, angina, and positive tryptase, which will help diagnose Kounis syndrome. However, the tryptase level was not measured for this patient. On the other hand, the presence of coronary vasospasm without thrombosis indicates Ischemic Heart Scombroid Syndrome (IHSS) [6, 12].

## 4 Conclusion

Although Kounis syndrome is not rare, the literature provides limited references to it. In clinical practice, doctors must recognize Kounis syndrome, as it requires prompt treatment decisions. Early identification and rapid management of Kounis syndrome, which involves anti‐ischemic, anti‐thrombotic, and anti‐allergic strategies, can significantly improve patient outcomes. Furthermore, coronary angiography may be considered for those patients.

## Author Contributions

**Abdelkarim Mohamed:** supervision, writing – original draft. **Obada Adel Alsakaji:** visualization, writing – original draft. **Muad Abdi Hassan:** visualization, writing – original draft, writing – review and editing. **Bassem Al Hariri:** supervision, writing – original draft.

## Ethics Statement

The study was conducted by the principles of the institutional ethical standards and the national research committee.

## Consent

Written informed consent was obtained from the patient for the publication of this case report and any accompanying images. This patient provided oral and signed written consent to use his clinical materials in this study.

## Conflicts of Interest

The authors declare no conflicts of interest.

## Data Availability

The authors have nothing to report.
